# Microbial Detoxification of Deoxynivalenol (DON), Assessed via a *Lemna minor* L. Bioassay, through Biotransformation to 3-epi-DON and 3-epi-DOM-1

**DOI:** 10.3390/toxins9020063

**Published:** 2017-02-13

**Authors:** Ilse Vanhoutte, Laura De Mets, Marthe De Boevre, Valdet Uka, José Diana Di Mavungu, Sarah De Saeger, Leen De Gelder, Kris Audenaert

**Affiliations:** 1 Laboratory of Environmental Biotechnology, Department of Applied Biosciences, Faculty of Bioscience Engineering, Ghent University, 9000 Ghent, Belgium; Ilse.Vanhoutte@UGent.be (I.V.); Laura.DeMets@UGent.be (L.D.M.); 2 Laboratory of Food Analysis, Department of Bioanalysis, Faculty of Pharmaceutical Sciences, Ghent University, 9000 Ghent, Belgium; Marthe.DeBoevre@UGent.be (M.D.B.); Valdet.Uka@UGent.be (V.U.); Jose.DianaDiMavungu@UGent.be (J.D.D.M.); Sarah.DeSaeger@UGent.be (S.D.S.); 3 Laboratory of Applied Mycology and Phenomics, Department of Applied Biosciences, Faculty of Bioscience Engineering, Ghent University, 9000 Ghent, Belgium; Kris.Audenaert@UGent.be

**Keywords:** deoxynivalenol (DON), *Lemna minor*, bioassay, biotransformation, detoxification, 3-epi-DON, 3-epi-de-epoxy-DON (3-epi-DOM-1)

## Abstract

Mycotoxins are toxic metabolites produced by fungi. To mitigate mycotoxins in food or feed, biotransformation is an emerging technology in which microorganisms degrade toxins into non-toxic metabolites. To monitor deoxynivalenol (DON) biotransformation, analytical tools such as ELISA and liquid chromatography coupled to tandem mass spectrometry (LC-MS/MS) are typically used. However, these techniques do not give a decisive answer about the remaining toxicity of possible biotransformation products. Hence, a bioassay using *Lemna minor* L. was developed. A dose–response analysis revealed significant inhibition in the growth of *L. minor* exposed to DON concentrations of 0.25 mg/L and higher. Concentrations above 1 mg/L were lethal for the plant. This bioassay is far more sensitive than previously described systems. The bioassay was implemented to screen microbial enrichment cultures, originating from rumen fluid, soil, digestate and activated sludge, on their biotransformation and detoxification capability of DON. The enrichment cultures originating from soil and activated sludge were capable of detoxifying and degrading 5 and 50 mg/L DON. In addition, the metabolites 3-epi-DON and the epimer of de-epoxy-DON (3-epi-DOM-1) were found as biotransformation products of both consortia. Our work provides a new valuable tool to screen microbial cultures for their detoxification capacity.

## 1. Introduction

Mycotoxins are secondary metabolites produced by fungi, posing serious risks to health and economy when present in food or feed products. In order to reduce these risks, pre-harvest crop management strategies have been introduced. Fungicides, well-considered crop rotation, turning tillage techniques, resistant varieties and biocontrol all contribute to reducing mycotoxins in the crop [1,2]. In addition, methods of downstream post-harvest processing such as sorting, dehulling and milling amongst others help to reduce the mycotoxin level in agricultural commodities [3]. Although implementing good agricultural and processing practices may diminish fungal infestation and mycotoxin production, full prevention of mycotoxin contamination is impossible to achieve. Therefore, detoxification strategies have been introduced as remediation tools for contaminated food and feed batches. Mycotoxin binders can be used. However, these can interact with other molecules in the gastro-intestinal tract of animals (e.g., medicines and antibiotics) [4]. Microbial and enzymatic degradation in which the mycotoxin molecules are effectively altered and thereby detoxified, pose a more attractive alternative [2,5]. 

The mycotoxin deoxynivalenol (DON), occurring worldwide, is produced by *Fusarium culmorum* and *Fusarium graminearum* in cereals (e.g., maize, wheat and barley) [6,7]. DON is a sesquiterpenoid trichothecene containing a 12,13-epoxide group, which is responsible for its toxicity by inhibiting protein synthesis [8,9]. Acute exposure can cause vomiting, nausea and diarrhea. Effects of chronic low-dose exposure are decreased weight gain, anorexia, decreased nutritional efficiency and altered immune function [10,11]. The European Commission has set a maximum level of DON for humans in unprocessed grains at 1.25 mg/kg; the guidance level for animals in feed is in general 8 mg/kg (dependent of type of feed or animal) [12,13]. DON is a recalcitrant molecule, resisting most downstream processing operations [14], and is not effectively removed from the matrix by binders [15]. Therefore, elimination of DON and other mycotoxins from contaminated matrices via microbial biotransformation might be a valuable emerging technology [16]. 

Biotransformation of DON by mixed cultures or isolates originating from different environmental sources has been reported. A large number of DON-degrading bacteria are found in rumen fluid or intestines, where DON is anaerobically transformed into de-epoxy-deoxynivalenol (DOM-1) [17,18,19,20,21,22,23]. Soil is also a promising source of DON-degrading organisms [24,25,26,27,28,29]. Bacterium E3-39, classified in the *Agrobacterium-Rhizobium* group, can transform DON into 3-keto-DON [29]. *Nocardioides* WSN05-2 and *Devosia mutans* 17-2-E-8 convert DON into 3-epi-DON in aerobic conditions [24,25], whereas *Citrobacter freundii* degrades DON into DOM-1 aerobically and anaerobically [26]. Besides soil, other microbial communities capable of DON biotransformation have been reported [28,30]. 

However, modification of a compound does not automatically entail detoxification [16,31], which is of course the ultimate goal. Metabolites can have residual or even heightened toxicity, which is often overlooked in biotransformation studies when using solely analytical tools [16]. For DON, some known derivatives have been tested on toxicity. In acetylated trichothecenes, loss of side groups on C4, C15 or C8 resulted in reduced protein synthesis inhibition [32]. However, 15-acetyl-deoxynivalenol (15-ADON) had a similar toxicity as the parent toxin DON, whereas 3-acetyl-deoxynivalenol (3-ADON) was less toxic than DON [33]. Toxicity tests have also been performed on metabolites of DON. It seems that 3-keto-DON is three to ten times less toxic than DON [29,34] and DOM-1 and 3-epi-DON are at least 50 times less toxic than DON [33,34,35]. When studying detoxification of mycotoxins, toxicity assays are crucial to assess toxicology. Animal trials can be performed but are expensive, time consuming and hampered by ethical issues. Cell culture-based systems [10,19,32,33,36,37,38] provide information about the metabolism of mycotoxins, but require a high workload (e.g., acquirement of cell lines, sterile work environment, long preparations and expensive reagents). Therefore, researchers have looked for alternative assays to estimate toxicity. Mycotoxins are known to induce adverse effects in many other organisms including birds, amphibians, arthropods, crustaceans, unicellular organisms, microorganisms and plants [39]. DON has been tested on (phyto)toxicity and relative toxicity towards other mycotoxins with several bioassays (e.g., *Arabidopsis thaliana*, wheat, *Lemna pausicostata*, *Chlamydomonas reinhardtii*, *Artemia salina* L (brine shrimp larvae), *Tetrahymena pyriformis* (ciliated protozoa) and (engineered) yeasts [40,41,42,43,44,45,46,47]. These bioassays are inexpensive, fast and require a lower workload.

In this work, a highly sensitive DON bioassay was developed and implemented to screen bacterial cultures for their detoxification capacity of DON and other trichothecenes using the aquatic macrophyte *Lemna minor* L. as an indicator organism. The goal was to develop a robust, inexpensive, highly sensitive and readily applicable high-throughput method to screen bacterial strains or enrichment cultures for their ability to biotransform and detoxify DON. The trichothecene DON is, in addition to being a mycotoxin, also a phytotoxin, and due to this feature, a plant-based bioassay can be used. *Lemna minor* L. is a well-known plant for use in bioassays and has been previously used to evaluate the biodegradation of herbicides [48] and to determine the toxicity of fumonisins [49,50,51]. To our knowledge, it was never used as an indicator organism for the toxicity of DON or trichothecenes. After development of the bioassay, a diverse set of matrices comprising rumen fluid, soil, digestate from an anaerobic digester and activated sludge from a water treatment plant, were used as inoculum for DON-degrading enrichment cultures. These cultures were analyzed on their detoxification capabilities with the bioassay and on their biotransformation capabilities with analytical tools. 

## 2. Results

### 2.1. Developing the Bioassay Using Lemna Minor

#### 2.1.1. Linearity

In order to assess the sensitivity of *Lemna minor* to DON, a wide concentration range was tested (0 (control)–0.1–0.5–1–5–10–50–100 mg/L DON). After 7 days, DON caused a reduction of 41% ± 12% in growth of *Lemna minor* at a concentration of 0.5 mg/L DON. Growth was completely inhibited at concentrations of 1 to 100 mg/L DON. In addition, an increase of bleached fronds was observed at 0.1 to 10 mg/L DON, while at concentrations of 10, 50 and 100 mg/L, 100% of the fronds were bleached. A correlation between the presence of DON and growth inhibition was further investigated for lower concentrations between 0 and 1 mg/L DON.

**Figure 1:**
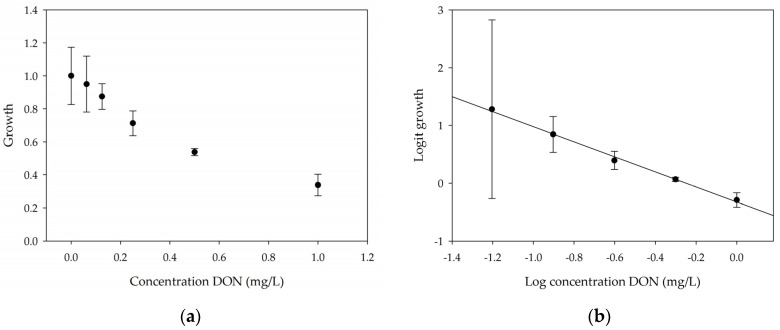

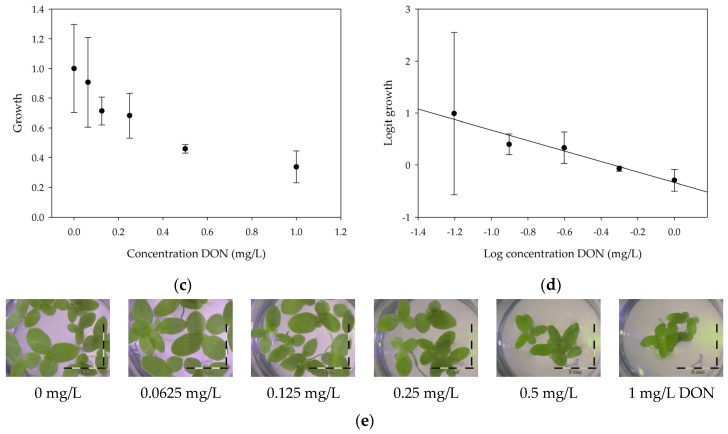
Growth of *Lemna minor* in response to deoxynivalenol (DON): (**a**) Data calculated based on number of fronds: correlation of growth and concentration DON (0–1 mg/L DON); (**b**) Data calculated based on number of fronds: correlation of logit growth and log concentration DON (0.0625–1 mg/L DON); (**c**) Data calculated based on frond area: correlation of growth and concentration DON (0–1 mg/L DON); (**d**) Data calculated based on frond area: correlation of logit growth and log concentration DON (0.0625–1 mg/L DON); (**e**) Response of *Lemna minor* after 7 days to increasing DON concentrations. Legend: black-white bar = 5 mm.

A calibration curve was set up with concentrations of 0, 0.0625, 0.125, 0.25, 0.5 and 1 mg/L DON starting from six fronds to assess the sensitivity of the bioassay (Figure 1e). The number of fronds (Figure 1a,b) and frond area (Figure 1c,d) are observed as growth parameters. For each parameter, a sigmoid correlation was found between the frond growth (Figure 1a,c) and the concentration of DON, confirming the conventional response of *Lemna minor* to growth-inhibiting compounds [52]. A log/logit transformation was carried out to obtain a linear relationship (Figure 1b,d). This transformation resulted in a good correlation between log(DON), and logit(growth_number of fronds_) and logit(growth_frond area_) with an R² of 0.996 (*p*-value < 0.001) and 0.947 (*p*-value = 0.005) respectively. 

From this tight correlation between *Lemna* growth reduction and DON concentration, it can be concluded that this bioassay is suitable as a tool to assess the toxicity mediated by DON. Next to frond growth and frond area, pulse amplitude-modulated chlorophyll fluorescence was also used to evaluate the impact of the toxin on the plant. After 12 h, a decrease in the quantum yield of Photosystem II (φ_PSII_) was observed, indicating that DON interferes with photosynthesis (Figure S1).

#### 2.1.2. Repeatability and Sensitivity

**Figure 2:**
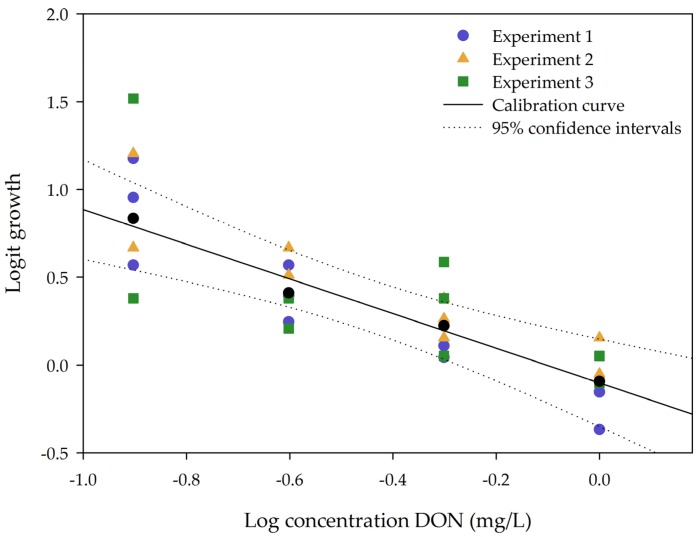
The calibration curve is expressed as the logit growth of number of fronds in function of the log concentration DON (mg/L). Data is shown from 0.125 to 1 mg/L DON. Triplicates of each experiment are illustrated in the same color.

To determine the variability of the impact of DON on plant growth, calibration curves (ranging from 0, 0.125, 0.25, 0.5 to 1 mg/L DON) were tested in triplicate on different days, performed by two people. The 95% confidence intervals were calculated for the main calibration curve. The results are shown in Figure 2. 

As seen in Figure 2, there is a high variation in response of the concentration at 0.125 mg/L DON. Some data points are located outside the 95% confidence interval. However, at higher concentrations, the variation within all three independent experiments is lower. The plant is significantly sensitive to DON at 0.250 mg/L: from a non-parametric Kruskal–Wallis test followed by a one-sided post-hoc Dunn’s test (α: 0.05) it could be concluded that a concentration of 0.250 mg/L DON (*n* = 9) significantly differs from the control. This result is in concordance with the chlorophyll fluorescence measurements carried out at 24 h after DON application (Figure S1). 

#### 2.1.3. Sensitivity of *Lemna Minor* towards Other *Fusarium* Mycotoxins

In order to assess the applicability of this bioassay to other trichothecenes and to zearalenone (ZEN), the bioassay was performed in triplicate on diacetoxyscirpenol (DAS), fusarenon X (FUS-X), T-2 toxin, HT-2 toxin, and nivalenol (NIV), which are trichothecene mycotoxins, and on the estrogenic mycotoxin ZEN at concentration level 1 mg/L. The plant *Lemna minor* was sensitive to DAS, FUS-X, T-2 and HT-2, but not to NIV and ZEN (Table 1). 

### 2.2. Implementing the Bioassay to Screen for DON Detoxification by the Enrichment Cultures

The obtained enrichment cultures (originating from a 6-week enrichment of rumen fluid, soil, digestate and activated sludge) were inoculated in “minimal incubation medium” (MMO) with 5 and 50 mg/L DON and incubated for four weeks at 30 °C and 100 rpm. The samples taken at four weeks of incubation were analyzed for the detoxification potential of the enrichment cultures (Figure S2). Sterilized culture filtrates were analyzed with the bioassay with a previous dilution step to an estimated 1 mg/L based on the amended DON-concentrations (5 and 50 mg/L) (Table 2). If a microbial community present in an enrichment culture was able to detoxify DON, we would expect the *Lemna* plants to show a better growth compared to the *Lemna* plants in control wells effectively exposed to 1 mg/L of DON. 

A high growth reduction is observed when *Lemna* plants are incubated with culture filtrate originating from enrichment cultures of rumen fluid and digestate. The growth reduction is similar to *Lemna* plants incubated with 1 mg/L of DON, indicating that no detoxification occurred. *Lemna* plants incubated with culture filtrate of the enrichment cultures of soil or activated sludge showed a similar growth compared to the control where no DON was added. These findings indicate that the enrichment cultures originating from soil and activated sludge contain some promising microorganisms capable of detoxifying DON aerobically within four weeks. 

These data were also confirmed with LC-MS/MS. No modification of DON occurred with digestate and rumen fluid cultures within four weeks, whereas soil and activated sludge cultures fully converted 50 mg/L DON. The same trend is observed for 5 mg/L. The cultures originated from soil and activated sludge could convert 100% ± 0% of 5 mg/L DON, whereas the cultures of digestate and rumen fluid only converted 11% ± 3% and 9% ± 9% of 5 mg/L DON, respectively. These findings have been statistically evaluated using a one-way ANOVA test followed by a Dunnett T3 test for pairwise multiple comparisons (α: 0.05). The samples of the control, digestate and rumen fluid are significantly different from the samples of activated sludge and soil. In addition, a quantitative determination was performed of known derivatives or metabolites of DON: 3-ADON, 15-ADON, deoxynivalenol-3-glucoside (DON-3G) and DOM-1. However, none of these molecules were found. 

**Figure 3:**
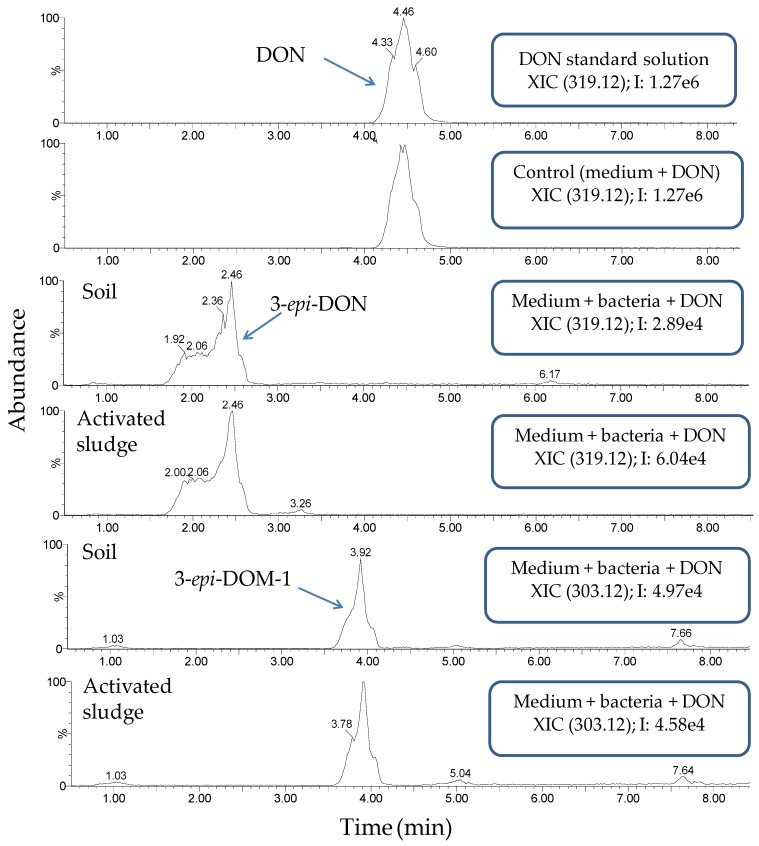
Extracted ion chromatograms (XICs) of the samples at 50 mg/L DON soil and activated sludge after four weeks, including the control (medium and DON). DON and 3-epi-DON were detected as both the protonated molecules and sodium adducts. Only the XICs for the sodium adducts are shown.

Subsequently, a more detailed investigation of the samples at 50 mg/L DON was performed by LC-high resolution MS (LC-HRMS) (Figure 3). The samples of digestate and rumen fluid showed a metabolite profile that was similar to the control (medium and DON). DON was still present and no novel entities could be detected. In contrast, investigation of the chromatograms of soil and activated sludge revealed two additional compounds, which were eluted before DON, i.e., at retention time (RT) 2.4 min and at RT 3.9 min. The compound at RT 2.4 min, with [M + H^+^] = 297.1333, was putatively assigned as 3-epi-DON (C_15_H_21_O_6_, mass error = −1.68 ppm), while the other biotransformation compound (RT 3.9 min, [M + H^+^] = 281.1392) was tentatively identified as 3-epi-DOM-1 (C_15_H_21_O_5_, mass error = 1.06 ppm). The identification of these compounds was confirmed by close examination of their chromatographic retention and fragmentation pattern (Figures S3 and S4) in light of data acquired for authentic standards of DON and DOM-1. The same fragment ions can be seen in the MS/MS spectra of each pair of the parent- and the epi-compounds. Interestingly, the intensities of the ions are different, as commonly observed with these types of isomers [53]. Our data corroborate previous studies that identified 3-epi-DON as biotransformation product of DON, and which showed that the former is eluted before the latter in reversed-phase chromatography [53,54,55]. Similarly, those studies also support our identification of 3-epi-DOM-1, in that this epimer was also eluted before the main compound DOM-1 (authentic standard).

### 2.3. Monitoring Detoxification (Bioassay) and Biotransformation (LC-MS/MS) by the Enrichment Cultures Soil and Activated Sludge through Time

In order to get a sense of DON detoxification and biotransformation kinetics exhibited by enrichment cultures of soil and activated sludge, we analyzed the supernatant of the intermediate samples taken at weeks 1, 2 and 3 of incubation (Figure S2). For DON detoxification, results from the bioassay are shown in Table 3, expressed as % growth, directly related to % detoxification. For DON biotransformation, results from the LC-MS/MS are shown in Table 4, expressed as % biotransformation. 

A stable concentration of DON is observed over time for the controls 5 mg/L (respectively 5 ± 1 mg/L, 5 ± 3 mg/L and 4 ± 1 mg/L for weeks 1, 2 and 3) and 50 mg/L DON (respectively 51 ± 11 mg/L, 48 ± 16 mg/L and 45 ± 6 mg/L for weeks 1, 2 and 3), indicating no abiotic influences on DON during the experiment. Analysis with the bioassay shows that exposure of each control supernatant (originating from the 5 mg/L and 50 mg/L control experiments, diluted to 1 mg/L) resulted in approximately 30% growth of *Lemna minor* (Table 3), which is in agreement with other experiments at 1 mg/L DON (Figure 1). 

Supernatant for soil enrichment culture starting at 5 mg/L DON results after one week in the same growth reduction as in the sterile DON control. However, after two and three weeks, 93% ± 3% and 86% ± 21% growth was detected, respectively (compared to the control without DON). This is in agreement with the LC-MS/MS analysis where DON was no longer detected after two weeks. Similar results were obtained starting from 50 mg/L DON. The activated sludge enrichment cultures displays other kinetics. At low concentrations (5 mg/L DON), DON was no longer detected with LC-MS/MS after week two and three. However, in week two and three, a slightly phytotoxic effect is still observed. At higher concentrations (50 mg/L DON), DON was again fully biotransformed after week two and three, whereas detoxification already occurred in week one. 

## 3. Discussion

In this study, we have developed and implemented a highly sensitive bioassay using *Lemna minor* L. for screening bacterial cultures on their DON detoxification capacities. To assess the sensitivity of *Lemna minor* L. to DON, a dose–response curve was established and a linear relation was found between logit (*Lemna* growth) and log (DON concentration). It should be mentioned that for the low concentrations 0.0625 and 0.125 mg/L DON, the variation in replicates was fairly high. Although we do not have any evidence, this might be due to small differences in the physiological fitness of the *Lemna* plants where some plants are inhibited by such low DON doses while others are not. This is in contrast to higher DON concentrations, for which plants are equally sensitive, resulting in very reproducible results. Therefore, the bioassay should be used to detect loss of toxicity starting from 0.25 mg/L DON. Growth of *Lemna minor* L. in response to DON was assessed by frond area and number of fronds. In the future, other response parameters might be included in the bioassay. We provide evidence that chlorophyll fluorescence might be a fast alternative and a sensitive parameter to implement in detoxification studies as previously shown for herbicides [48]. Measuring the conductivity of the medium might be a fourth parameter to determine the electrolyte release [40]. Subsequently, the usability of the *Lemna* bioassay was tested for other trichothecenes and for the estrogenic mycotoxin ZEN. ZEN and NIV seemed to be non-toxic for *Lemna minor*, in contrast to DON, T-2 toxin, HT-2 toxin, DAS and FUS-X. Therefore, the bioassay can be used as a cheap bio-tool for screening the phytotoxicity of DON, other tricothecenes and DON derivatives. 

The sensitivity of the bioassay was statistically evaluated, where a concentration of 0.25 mg/L DON was found to be significantly different from the control. Between 1 and 100 mg/L DON, complete growth inhibition and leaf necrosis occurred, whereas at 0.25 until 1 mg/L growth reduction occurred. In our bioassay, 34% ± 6% growth was observed at 1 mg/L DON after 7 days resulting in a highly sensitive bioassay. In Table 5, our assay is compared to other existing bioassays, including information about advantages, drawbacks and limitations, as well as the workload, time requirements and applicability of the bioassays. The various bioassays are ranged from lowest to highest sensitivity for DON.

As seen in Table 5, the sensitivity of most bioassays ranged from a concentration of 30 down to 3 mg/L DON. Only the ciliated protozoa *Tetrahymena pyriformis* was also reported as a very sensitive organism for DON with 0.6 mg/L DON as minimum active dose. However, our bioassay can detect analyte concentrations as low as 0.25 mg/L DON, which to our knowledge has not been reported before. 

Implementing bioassays in biotransformation experiments is of great importance because the toxicity of metabolites is often overlooked in screening assays for new promising microbial strains. Several examples in literature are available showing that mycotoxin modification does not always result in mycotoxin detoxification, which illustrates the necessity and benefits of bioassays as developed in this study. For aflatoxin B1, the metabolite aflatoxin M1 is a commonly known metabolite which is categorized as possibly carcinogenic to humans (Group 2B) by the International Agency for Research on Cancer [57]. The same accounts for ZEN, for which several metabolites have been found with similar or even more estrogenic activity [16,58]. Furthermore, this problem is nicely illustrated by the following ‘detoxification’ reaction of 3-ADON which is first converted to DON as an initial step which leads to an increased toxicity, subsequently followed by de-epoxidation into the non-toxic metabolite DOM-1 [44]. 

Enrichment cultures were evaluated for their DON detoxification capacity through the *Lemna minor* bioassay. The microbial communities originating from digestate and rumen fluid did not show this capability, which is not entirely surprising as they are adapted to an anaerobic environment, whereas the enrichment and the biotransformation experiment were performed in the presence of oxygen. The cultures obtained from soil and activated sludge showed a clear detoxification through time with the total disappearance of 5 and 50 mg/L DON after two weeks, as assessed by LC-MS/MS. Soil has already been reported as a source for DON-transforming microorganisms [24,25,26,27,28,29], but to our knowledge, this is not the case for activated sludge from a water treatment plant.

LC-HRMS analysis of the soil and activated sludge cultures after 4 weeks of biotransformation revealed two DON metabolites, namely 3-epi-DON and 3-epi-DOM-1. It has been shown that 3-epi-DON is less toxic than DON [34,35] and has been reported to be formed by several bacteria belonging to the genera *Devosia* and *Nocardioides* [25,28,59]. It has been shown that de-epoxidation of DON to DOM-1 already lowers toxicity [33,35], so although toxicological studies for 3-epi-DOM-1 are not available yet, one can assume that this compound is also less toxic than the parent mycotoxin DON as it is its de-epoxidized and epimerized form. To date, 3-epi-DOM-1 has never been reported as a microbial transformation product of DON. 

The observation that the disappearance of DON, as detected by LC-MS/MS, after two weeks in soil and activated sludge cultures, does not completely match the detoxification data obtained via the *Lemna minor* bioassay, points to differences in kinetics of DON transformation between soil and activated sludge cultures and/or small differences in toxicity between the detoxification products 3-epi-DON and 3-epi-DOM-1. Therefore, an in-depth study on the kinetics of DON transformation by these enrichment cultures will be a very interesting step in further research. Thereby, the bioassay presented here will be an indispensable tool in assessing detoxification and therefore complement purely analytical tools in this and other biotransformation studies.

## 4. Materials and Methods

### 4.1. Sources

Rumen fluid was sampled via a rumen fistula of three sheep at the experimental farm of Ghent University. Soil originated from a monoculture corn field in Nazareth, Belgium. Digestate came from an anaerobic digester provided by Innolab (Ghent, Belgium). Activated sludge originated from a municipal water treatment plant (Gavere, Belgium). The aquatic plant *Lemna minor* was maintained in an aquarium at room temperature under a light regime of 16 h/8 h (light/dark) illuminated with a Solar Lux 26 Watt lamp (Aquatic nature, Ghent, Belgium). The aquarium contained 40 L of tap water each week supplemented with 1 mL of macro elements stock solution containing 60 g/L KNO_3_, 12 g/L KH_2_PO_4_ and 32 g/L K_2_SO_4_. 

### 4.2. Source Material Preparation

Before use as inoculants for the enrichment experiments, the microbial community sources were prepared. Rumen fluid was directly used as inoculum. A soil sample weighing 20 g was suspended in 25 mL physiological water (8.5 g/L NaCl in distilled water) and added to a stomacher for 1 minute at low speed in order to homogenize the sample and separate the liquid phase. Digestate (30 mL) was transferred into a stomacher for 2 min at normal speed to homogenize the sample and separate the liquid phase. An aliquot of activated sludge sample was mixed homogeneously and added to a 1.5-mL recipient. The floc structure of activated sludge was broken down through pipetting up and down with a sterile syringe and needle releasing the bacteria into suspension. 

### 4.3. Standards

The individual mycotoxin solid calibration standards (1 mg) of DON, 3-ADON, 15-ADON, DOM-1, NIV and zearalanone (ZAN, internal standard) were purchased from Sigma-Aldrich (Bornem, Belgium). DON-3G (50.2 ng/µL, in acetonitrile) was purchased from Biopure Referenzsubstanzen (Tulln, Austria). 

All mycotoxin solid standards for LC-MS/MS analysis were dissolved in methanol (1 mg/mL), and were storable for a minimum of 1 year at −18 °C [60]. DON-3G was kept at 4 °C. The working solutions of DON, 3-ADON, 15-ADON, DOM-1 and NIV (10 ng/µL) were prepared in methanol, and stored at −18 °C, while DON-3G (10 ng/µL) was dissolved in acetonitrile (4 °C, monthly renewed).

DON standard for the bioassay and the enrichment protocol was dissolved in dimethyl sulfoxide (DMSO) at a concentration of 1 mg/mL and stored at −18 °C. Working solutions of 100 and 10 mg/L DON were prepared in “*Lemna* medium” or “minimal incubation medium” (MMO) and stored at −18 °C. 

### 4.4. Enrichment for DON-Degrading Microorganisms

Screening of microorganisms with respect to DON biotransformation capacity was performed by adding the four inoculants at 0.1% in 50 mL sterile MMO. This medium is based on Stanier medium [61] and contained 1.4 g/L Na_2_HPO_4_, 1.4 g/L KH_2_PO_4_, 0.3 g/L (NH_4_)_2_SO_4_, 98.5 mg/L MgSO_4_, 5.9 mg/L CaCl_2_.H_2_O, 3.2 mg/L Na_2_EDTA, 2.8 mg/L FeSO_4_.7H_2_O, 1.2 mg/L ZnSO_4_.7H_2_O, 1.7 mg/L MnSO_4_.H_2_0, 0.4 mg/L CuSO_4_.5H_2_O, 0.2 mg/L CoCl_2_.6H_2_O and 0.1 mg/L (NH_4_)_6_Mo_24_.4H_2_O. The liquid medium was supplemented with 50 mg/L DON as the only carbon source. The enrichment was incubated for 6 weeks at 100 rpm and 30 °C. Controls without bacteria and without DON were included. After the incubation period, enrichment cultures were obtained and archived at −80 °C in 20% *v*/*v* glycerol. 

### 4.5. Biotransformation Experiment

For the biotransformation experiment, precultures were made in MMO medium. The obtained enrichment cultures (stored at −80 °C) were added into 10 mL MMO medium at 1% with 5 or 50 mg/L DON. The aliquots were incubated at 30 °C and 100 rpm. Microorganism growth of the precultures was monitored with a spectrophotometer, measuring the optical density at 600 nm. The precultures were used as an inoculum for the biotransformation experiment at an optical density value of 1. Subsequently, 2% of each preculture inoculum was added with 5 or 50 mg/L DON into 10 mL MMO medium. The experiment was performed in triplicate. Controls are included (MMO medium with 5 or 50 mg/L DON without addition of microbial source and MMO medium with microbial sources without DON). The enrichment cultures were incubated for four weeks at 30 °C and 100 rpm. Samples were taken weekly and stored at −18 °C. 

### 4.6. Bioassay Protocol

A mineral growth medium for *Lemna minor* was prepared according to Megateli et al. [62]. A sterile 24-well plate was used to grow *Lemna minor* in “*Lemna* medium”, in a total volume of 2 mL per well. Starting from six fronds, the plants were incubated in triplicate for 7 days in a growth chamber (16 h light exposure, 22 °C). For each analysis of samples, a calibration curve was included (0, 0.0625, 0.125, 0.25, 0.5 and 1 mg/L DON). Samples were filter sterilized using a sterile filter and syringe. Each sample was diluted to the range of calibration curves. Samples starting from 5 mg/L DON were diluted 5 times; samples starting from 50 mg/L DON were diluted 50 times resulting in a final concentration of 1 mg/L in the *Lemna* bioassay. After 7 days of incubation, the plates were analyzed based on frond growth. The number of fronds are counted with a microscope (Stereo IX). Photos are made of each well with the microscope and frond area is calculated with the program APS Assess. 

### 4.7. Detection of DON and Its Possible Metabolites by LC-MS/MS

#### 4.7.1. Sample Preparation

One milliliter of MMO medium was weighed in a glass tube (10 mL). A calibration curve was set up by spiking three blank medium samples with DON, NIV, 3-ADON, 15-ADON, DOM-1 and DON-3G (10 ng/µL, range 100 µg/L–200 µg/L–400 µg/L). The stock solution (25 µL) of the internal standard ZAN (10 ng/µL, 250 ng on sample) was added to both calibrators as unknowns. Samples were vortexed (Labinco BV, Breda, The Netherlands) for 2 min. The medium samples were evaporated until dryness under nitrogen at 60 °C using the TurboVap^®^ LV (Biotage, Dusseldorf, Germany), and redissolved in 100 µL of injection solvent (methanol/water/acetic acid (41.8/57.2/1, *v*/*v*/*v*) with 5 mM of ammonium acetate). Finally, the redissolved sample was vortexed for 3 min. Prior to LC-MS/MS analysis, the samples were collected in an Ultrafree-MC centrifugal device (0.22 µm, Millipore, Bedford, MA, USA) and centrifuged for 10 min at 10,000 g.

#### 4.7.2. LC-MS/MS Analysis

LC-MS/MS analysis was performed using a Waters Acquity HPLC system coupled to a Quattro Micro mass spectrometer (Waters, Milford, MA, USA) equipped with an electrospray interface (ESI) by injecting a volume of 20 µL. Chromatographic separation was performed using an Acquity HPLC Symmetry C18 column (5 µm, 150 mm × 2.1 mm) and a guard column of the same material (10 mm × 2.1 mm i.d.) (Waters, Milford, MA, USA). The column was kept at 60 °C, and the temperature of the autosampler was set at 10 °C. A mobile phase consisting of variable mixtures of mobile phase A (water/methanol/acetic acid, 94/5/1 (*v*/*v*/*v*), 5 mM ammonium acetate) and mobile phase B (methanol/water/acetic acid, 97/2/1 (*v*/*v*/*v*), 5 mM ammonium acetate) was used at a flow rate of 0.3 mL/min with a gradient elution program. The gradient elution started at 99% mobile phase A with a linear decrease to 35% in 7 min. The next 4-min mobile phase A decreased to 25%. An isocratic period of 100% mobile phase B started at 11 min for 2 min. Initial column conditions were reached at 23 min using a linear decrease of mobile phase B, and over 5 min mobile phase A was used to recondition the column. The duration of each HPLC run was 28 min. The mass spectrometer was operated in the positive electrospray ionization (ESI^+^) mode. MS parameters were as follows: ESI source block and desolvation temperatures: 150 and 350 °C, respectively; capillary voltage: 3.2 kV; argon collision gas: 1.15 × 10^−2^ mbar; cone nitrogen and gas flow: 50 L/h and 800 L/h, respectively. The data acquisition was performed using selected reaction monitoring (SRM, Table 6). Masslynx^TM^ version 4.1 and Quanlynx^®^ version 4.1. software (Waters, Milford, MA, USA) were used for data acquisition and processing.

The analytical method was validated according to Commission Decision 2002/657/EC [63], and all validation parameters met the criteria mentioned. Validation data for DON is shown in Table S1. Limit of detection (LOD) and limit of quantification (LOQ) ranged from 30 µg/L to 45 µg/L, and 61 µg/L to 89 µg/L, respectively.

#### 4.7.3. LC-HRMS Analysis

Chromatographic separation was achieved on an ACQUITY UPLC I-class FTN system (Waters, Manchester, UK), using a ZORBAX RRHD Eclipse Plus C18 (1.8 µm, 2.1 mm × 100 mm). The mobile phase consisted of H_2_O:MeOH (99:1, *v*/*v*) containing 0.05% HCOOH and 5 mM HCOONH_4_ (solvent A) and MeOH (solvent B). A gradient elution program was applied as follows: 0–0.5 min: 5% B, 0.5–20 min: 5%–95% B, 20–21 min: 95% B, 21–24 min: 95%–5% B, 24–28 min: 5% B. The flow rate was 0.3 mL/min. The column temperature was set at 40 °C and the temperature of the autosampler was 10 °C. Five microliters of the sample was injected.

HRMS analyses were performed using a hybrid quadrupole (Q) orthogonal acceleration time-of-flight (TOF) high-definition mass spectrometer, the Synapt G2-Si HDMS (Waters), equipped with an electrospray ionization (ESI) source. Data were acquired as positive ion (ESI^+^) polarity runs in resolution mode (> 20000 FWHM). The MS parameters were as follows: capillary voltage 2.8 kV; sample cone voltage 30 V; source temperature 150 °C; desolvation gas flow 800 L/h at a temperature of 550 °C and cone gas flow 50 L/h. Nitrogen was used as the desolvation and cone gases. Argon was employed as the collision gas at a pressure of 9.28 × 10^−3^ mbar. The instrument was calibrated using sodium formate clusters. During the MS analysis, a leucine-enkephalin solution (200 pg/µL) was continuously infused into the mass spectrometer at a flow rate of 20 μL/min via the lockspray interface, generating the reference ion ([M + H]^+^ = 556.2771) used for mass correction. Mass spectra were collected in continuum mode from *m*/*z* 50 to 1200 with a scan time of 0.1 s, an inter-scan delay of 0.01 s and a lockspray frequency of 20 s. A data-dependent acquisition (DDA) mode was implemented to obtain the simultaneous acquisition of exact mass data for the precursor and fragment ions. The top five ions were selected for MS/MS from a single MS survey scan. The scan time for MS/MS was 0.2 s. The collision energy in the trap cell was ramped from 10/15 V (low mass, start/end) up to 60/150 V (high mass, start/end). Instrument control and data processing were carried out using Masslynx 4.1 software (Waters).

### 4.8. Data Processing

For developing the bioassay, data was processed for the number of fronds, as well as for frond area. For each calibration curve, the growth was calculated with formula (1) and (2). Growth is plotted as function of concentration DON (mg/L). Subsequently, logit of growth was calculated (3) and plotted in function of logarithm of concentration DON (mg/L) to obtain a linear correlation. In every step, standard deviations were determined. These data were plotted with Sigmaplot 13. The regression coefficients and corresponding *p*-values were calculated with Sigmaplot 13 with linear regression.

(1) $$Growth_{number\;of\;fronds}=\frac{number\;of\;fronds}{number\;of\;fronds_{control}},$$

(2) $$Growth_{frond\;area}=\frac{frond\;area}{frond\;area_{control}},$$

(3) $$Logit\;growth=Log\frac{growth}{1-growth},$$

The three calibration curves used for evaluating the repeatability were also plotted in Sigmaplot 13, together with the 95% confidence intervals. 

For processing data of the bioassay samples, growth was calculated and expressed in % relative to the control. For processing data of LC-MS/MS, % biotransformation of treatments was calculated relative to the corresponding control at the same time point when the sample was taken. Data was statistically analyzed via a one-way ANOVA test or a non-parametric Kruskal–Wallis test followed by a Dunnett T3 test or Dunn’s test for pairwise multiple comparisons (α: 0.05) in SPSS Statistics 23. Standard errors were given as ±.

## Figures and Tables

**Table 1:** Sensitivity of *Lemna minor* towards other *Fusarium* mycotoxins.

Mycotoxin	% Growth at 1 mg/L Mycotoxin
DAS	26 ± 4 ^b^
DON	34 ± 6 ^b^
FUS-X	44 ± 12 ^b^
T-2 toxin	52 ± 12 ^b^
HT-2 toxin	54 ± 15 ^b^
ZEN	90 ± 42 ^a^
NIV	92 ± 11 ^a^
Control	100 ± 7 ^a^

^a,b^ Statistically analyzed via a non-parametric Kruskal–Wallis test followed by a one sided post-hoc Dunnett’s test (α: 0.05). DAS: diacetoxyscirpenol; FUS-X: fusarenon X; ZEN: zearalenone; NIV: nivalenol.

**Table 2:** Screening of enrichment cultures (rumen fluid, soil, digestate and activated sludge) after four weeks of incubation at 0, 5 and 50 mg/L DON, analyzed with the bioassay.

Concentration DON (mg/L)		Rumen fluid	Soil	Digestate	Activated sludge
0 mg/L DON		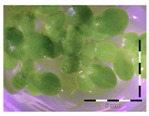 101 ± 9% ^a^	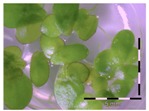 93 ± 6% ^a^	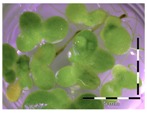 96 ± 4% ^a^	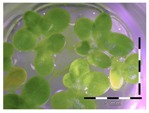 104 ± 10% ^a^
5 mg/L DON ^1^	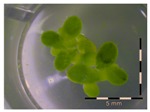	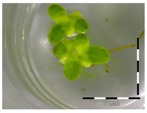 28 ± 0% ^b^	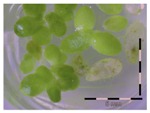 83 ± 6% ^a^	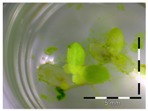 34 ± 5% ^b^	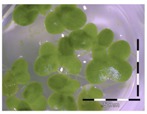 99 ± 13% ^a^
50 mg/L DON ^1^	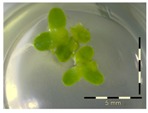	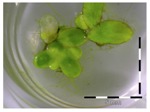 36 ± 2% ^b^	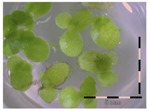 87 ± 10% ^a^	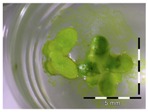 43 ± 4% ^b^	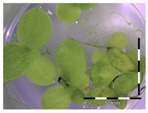 83 ± 4% ^a^

Growth (%) is mentioned below each figure. Legend: black-white bar = 5 mm. ^a,b^ Statistically analyzed via a non-parametric Kruskal–Wallis test followed by a one sided post-hoc Dunn’s test (α: 0.05). ^1^ Controls 5 and 50 mg/L DON diluted to 1 mg/L DON (5 and 50 times respectively), as well as the samples.

**Table 3:** Detoxification kinetics of 5 and 50 mg/L DON by enrichment cultures (soil and activated sludge) assessed through the *Lemna minor* bioassay.

Matrix	Growth of *Lemna minor* (%) after exposure to culture supernatant from the DON detoxification experiment after x weeks assessed by the bioassay		
	After 1 week of detoxification	After 2 weeks of detoxification	After 3 weeks of detoxification
**0 mg/L DON**			
Sterile control			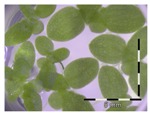 100 ± 4 ^a^
**5 mg/L DON**			
Sterile control ^1^	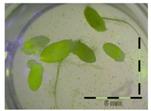 30 ± 2 ^b^	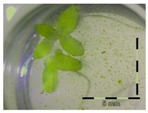 32 ± 1 ^b^	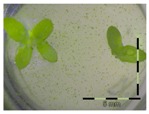 30 ± 2 ^b^
Soil	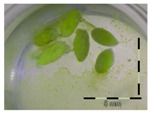 32 ± 6 ^b^	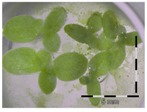 93 ± 3 ^a^	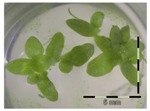 86 ± 21 ^a^
Activated sludge	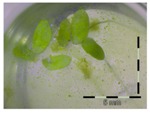 33 ^2^	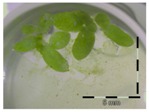 53 ± 4 ^b^	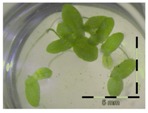 69 ± 4 ^b^
**50 mg/L DON**			
Sterile control ^1^	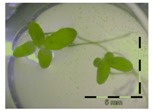 35 ± 5 ^b^	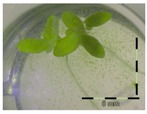 31 ± 4 ^b^	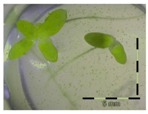 32 ± 3 ^b^
Soil	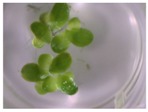 47 ± 1 ^b^	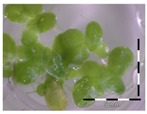 88 ± 4 ^a^	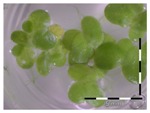 90 ± 3 ^a^
Activated sludge	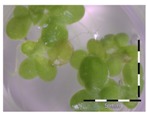 99 ± 1 ^a^	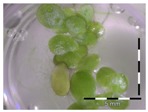 78 ± 15 ^a^	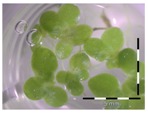 83 ± 3 ^a^

Legend: black-white bar = 5 mm. ^a,b^ Statistically analyzed via a non-parametric Kruskal–Wallis test followed by a one sided post-hoc Dunn’s test (α: 0.05). ^1^ Controls 5 and 50 mg/L DON diluted to 1 mg/L DON (5 and 50 times respectively), as well as the samples. ^2^ Sample activated sludge at 5 mg/L DON in week one exceptionally not in triplicate.

**Table 4:** Biotransformation kinetics of 5 and 50 mg/L DON by soil and activated sludge enrichment cultures assessed by LC-MS/MS.

Matrix	DON Biotransformation (%) in the Culture Supernatant from the DON Biotransformation Experiment after x Weeks Assessed by LC-MS/MS Analysis		
	After 1 Week of Biotransformation	After 2 Weeks of Biotransformation	After 3 Weeks of Biotransformation
**5 mg/L DON**			
Sterile control	0 ± 27 ^b^	0 ± 63 ^1,b^	0 ± 20 ^b^
Soil	56 ± 15 ^b^	100 ± 0 ^a^	100 ± 0 ^a^
Activated sludge	72 ± 2 ^a^	100 ± 0 ^a^	100 ± 0 ^a^
**50 mg/L DON**			
Sterile control	0 ± 22 ^b^	0 ± 32 ^b^	0 ± 14 ^b^
Soil	28 ± 6 ^b^	100 ± 0 ^a^	100 ± 0 ^a^
Activated sludge	68 ± 14 ^a^	100 ± 0 ^a^	100 ± 0 ^a^

^a,b^ Statistically analyzed via a non-parametric Kruskal–Wallis test followed by a one-sided post-hoc Dunn’s test (α: 0.05). ^1^ Sample control 5 mg/L DON in week two exceptionally in duplicate.

**Table 5:** Comparison of bioassays used for assessing DON toxicity.

Organism [Reference]	Application	Characteristics		
		Sensitivity	Time	Workload
Brine shrimp larvae *Artemia salina* L. [47,56]	General screening for trichothecenes in grains	600–1200 ng/disc (~30–60 mg/L) DON → 50% of mortality	30 h of preparation, 30 h of incubation	Preparation of larvae (including separation eggs) * Disc screening method: addition of 20 µL/disc toxin in a well with addition of 2 drops (±100 µL) of a suspension of larvae * Measuring mortality (counting immobile larvae under microscope, killing larvae, counting total number)
Unicellular algae *Chlamydomonas reinhardtii* [42]	* Testing cloned genes (resistance to trichothecenes) * Comparing trichothecenes with C3-OH group with their acetylated derivatives	25 mg/L DON → clear toxic effect	8 days	Preparation of preculture Measuring growth (haemocytometer), cell viability (plating), calculating number of culture doublings
Yeast *Klyveromyces marxianus* [44]	Screening DON-degrading organisms	23 mg/L DON → 50% growth inhibition 300 mg/L DOM-1 → no growth inhibition	Overnight preparation, 22 h of incubation	Preparation of preculture Incubation of sterile supernatants Measuring optical density at 650 nm
Engineered baker’s yeast [41]	* Use as a bioassay indicator organism * Screening for DON-detoxifying bacteria	5 mg/L DON → 50% growth inhibition	16 h	Preparation yeast culture * Agar diffusion test * Measuring optical density at 620 nm
*Arabidopsis thaliana* [45,46]	Studying the phytotoxic action of trichothecenes	* 10 µM (~3 mg/L) DON → no inhibition of seed germination, inhibition of root growth * 23.0 ± 6.8 µM (~6,8 ± 2 mg/L) DON → mortality of 50% of leaves	* 3 days of preparation, 3 days of incubation * 3 weeks of preparation, 7 days of incubation	* Preparation of seeds Investigation of growth and morphology * Preparation of 3-week-old plants Leave protocol: investigation of shriveling, chlorosis, death
Wheat plants [46]	Studying the phytotoxic action of trichothecenes	>15 µM (~4.5 mg/L) DON → inhibition of root elongation of wheat plants	3 days of preparation, 3 days of incubation	Preparation of seeds Investigation of growth and morphology
*Lemna pausicostata* [40]	Screening trichothecenes for bioherbicides	10 µM (~3 mg/L DON) → 56.0% ± 5.7% growth	72 h	No need for preparation Measuring electrolyte release (conductivity), growth inhibition, chlorophyll reduction
Ciliated protozoa *Tetrahymena pyriformis* [43]	* Testing toxicity of mycotoxins * Screening of cereals	0.6 mg/L DON → minimum active dose	Preparation of heat shocks, 150 min incubation	Preparation heat shocks for good division synchrony, requires good heating and cooling devices Measuring delay between start of division in control and toxin-treated culture, counting number of cells
*Lemna minor* L.	* Screening for phytotoxicity of DON and other trichothecenes * Screening for DON-degrading organisms	1 mg/L DON → 34% ± 6% growth 0.250 mg/L → minimum active dose (significantly different)	* 7 days * 12 h with chlorophyll fluorescence	No need for preparation Measuring number of fronds, frond area, chlorophyll fluorescence

**Table 6:** Selected reaction monitoring (SRM) transitions for the analyzed mycotoxins in liquid medium.

Analyte	Precursor Ion (*m*/*z*)	Molecular Ion	ConeVoltage (V)	Product Ions (*m*/*z*)	Collision Energy (eV)
**NIV**	313.1	[M + H]^+^	26	125.0 *	13
				205.0	12
**DON**	297.1	[M + H]^+^	26	231.2 *	15
				249.2	10
**DOM-1**	281.0	[M + H]^+^	26	109.1 *	19
				137.0	15
**3-ADON**	339.0	[M + H]^+^	28	203.2 *	12
				231.2	13
**15-ADON**	339.0	[M + H]^+^	26	261.0 *	10
				321.2	10
**DON-3G**	476.1	[M + NH_4_]^+^	15	248.6 *	18
				296.9	12
**ZAN (IS)**	321.0	[M + H]^+^	26	189.2 *	19
				303.3	13

* quantifier ion. DOM-1: de-epoxy-deoxynivalenol; 3-ADON: 3-acetyl-deoxynivalenol; 15-ADON: 15-acetyl-deoxynivalenol; DON-3G: deoxynivalenol-3-glucoside; ZAN: zearalanone.

## Supplementary Materials

The following are available online at www.mdpi.com/2072-6651/9/2/63/s1, Figure S1: A dose–response curve of chlorophyll fluorescence in *Lemna minor* at 12 h after exposure to different DON concentrations, Figure S2: Scheme of experimental setup of enrichment and biotransformation experiment, Figure S3: MS/MS spectra of DON and 3-epi-DON, Figure S4: MS/MS spectra of DOM-1 and 3-epi-DOM-1, Table S1: Validation parameters for DON.
