# Supplementary material for: Microbial Detoxification of Deoxynivalenol (DON), Assessed via a Lemna minor L. Bioassay, through Biotransformation to 3-epi-DON and 3-epi-DOM-1
Source: Toxins (Basel). 2017 Feb 13;9(2):63. doi: 10.3390/toxins9020063 (PMC5331442; doi:10.3390/toxins9020063)
Supplement: Supplementary file 1 [file toxins-09-00063-s001.pdf]

Microbial Detoxification of Deoxynivalenol (DON), Assessed via a *Lemna minor* L. Bioassay, through Biotransformation to 3-epi-DON and 3-epi-DOM-1

Ilse Vanhoutte, Laura De Mets, Marthe De Boevre, Valdet Uka, José Diana Di Mavungu, Sarah De Saeger, Leen De Gelder and Kris Audenaert

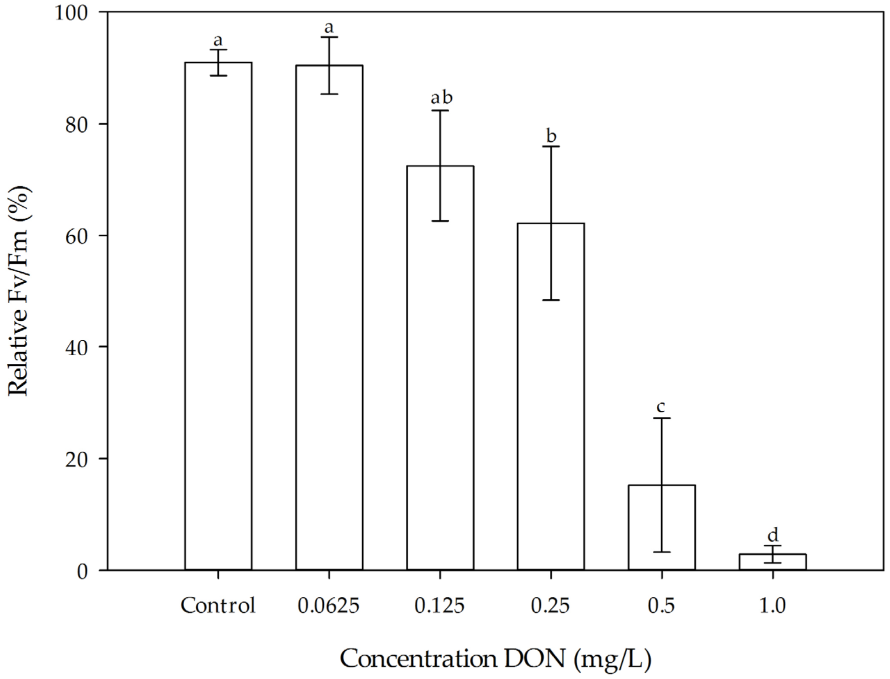

Figure S1. A dose response curve of chlorophyll fluorescence in *Lemna minor* at 12 h after exposure to different DON concentrations. The graph depicts relative chlorophyll fluorescence Fv/Fm values (Fm = maximum fluorescence and Fv = variable fluorescence (Fm-Fo)) in function of increasing DON concentrations. The data are expressed relatively to the surface of the *Lemna minor* fronds. Data were analyzed using a non-parametric Kruskal-Wallis test followed by a Dunn's test for pairwise multiple comparisons (α: 0.05). The chlorophyll fluorescence in *Lemna minor* plants was measured using a pulse amplitude modulated fluorometer.

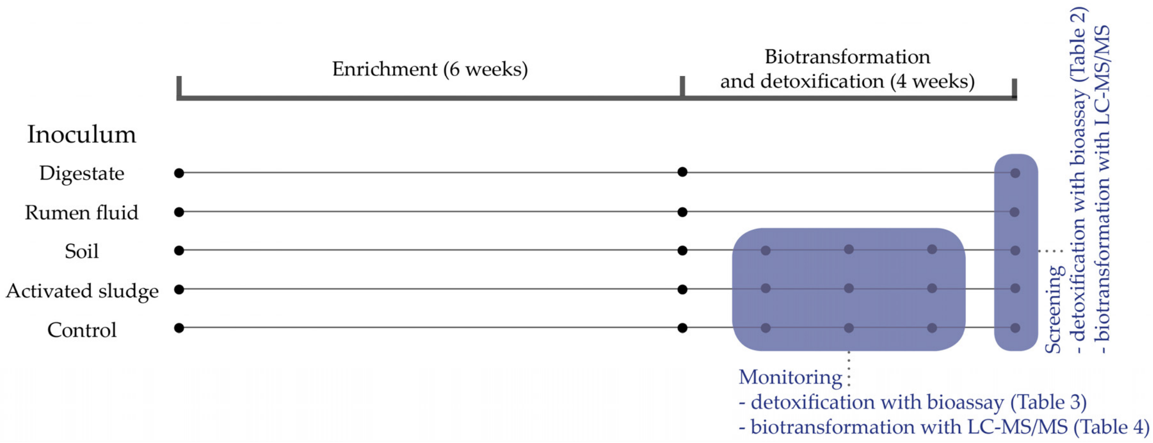

Figure S2. Scheme of experimental setup of enrichment and biotransformation experiment. In 2.2, screening of detoxification potential of enrichment cultures are determined with the bioassay at week 4 (Table 2). These results have been confirmed with LC-MS/MS. In 2.3, detoxification and biotransformation are assessed respectively with the bioassay (Table 3) and LC-MS/MS (Table 4) at week 1, 2 and 3.

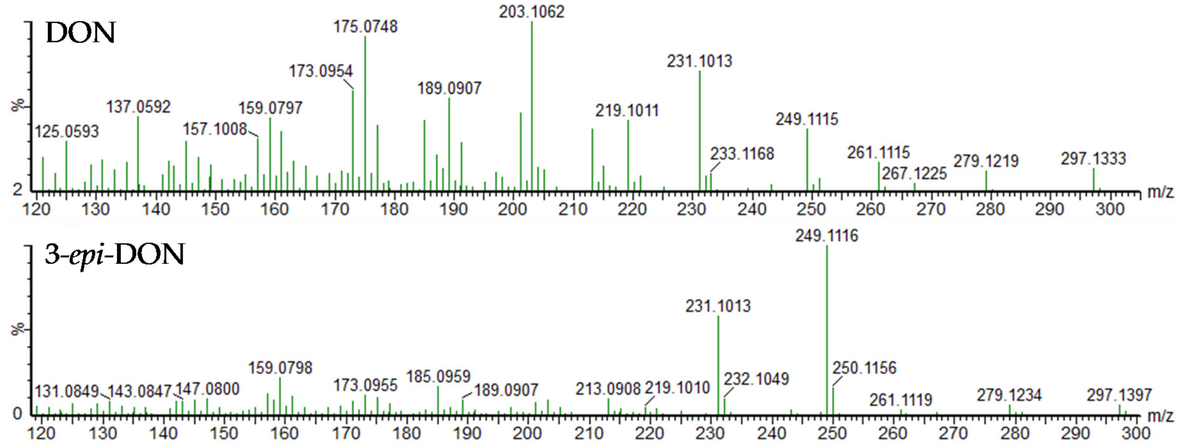

Figure S3. MS/MS spectra of DON and 3-epi-DON.

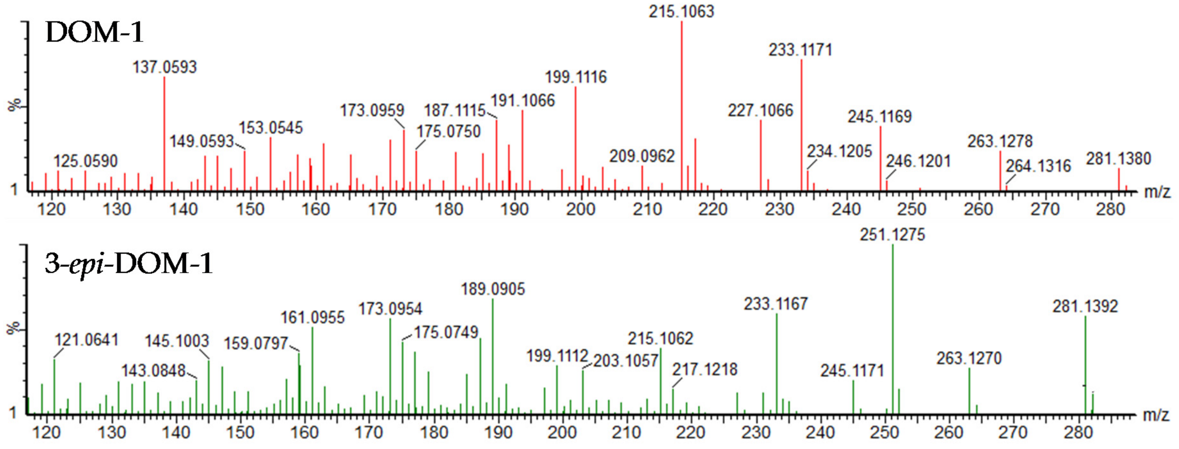

Figure S4. MS/MS spectra of DOM-1 and 3-epi-DOM-1.

Table S1. Validation parameters for DON.

Parameter	Value	Parameter	Value
**LOD (µg/kg)**	33	**Rep$_l$ (%)**	36
**LOQ (µg/kg)**	65	**Rep$_m$ (%)**	8
**CCα (µg/kg)**	18	**Rep$_h$ (%)**	5
**CCβ (µg/kg)**	27	**Repr$_l$ (%)**	7
**AR$_l$ (%)**	99	**Repr$_m$ (%)**	8
**AR$_m$ (%)**	101	**Repr$_h$ (%)**	3
**AR$_h$ (%)**	98		
**U$_l$ (%)**	46.23		
**U$_m$ (%)**	24.16		
**U$_h$ (%)**	10.57		

LOD = limit of detection; LOQ = limit of quantification; CCα = decision limit; CCβ = detection capability; AR= apparent recovery at low concentration (AR$_l$ = 20 µg/kg), medium concentration (AR$_m$ = 100 µg/kg) and high concentration (AR$_h$ = 200 µg/kg); Rep= repeatability at low concentration (Rep$_l$ = 20 µg/kg), medium concentration (Rep$_m$ = 100 µg/kg) and high concentration (Rep$_h$ = 200 µg/kg); Repr = within-laboratory reproducibility at low concentration (Repr$_l$ = 20 µg/kg), medium concentration (Repr$_m$ = 100 µg/kg) and high concentration (Repr$_h$ = 200 µg/kg); U= measurement uncertainty at low concentration (U$_l$ = 20 µg/kg), medium concentration (U$_m$ = 100 µg/kg) and high concentration (U$_h$ = 200 µg/kg).
